# Enhanced Electrochemical Characteristics of the Glucose Oxidase Bioelectrode Constructed by Carboxyl-Functionalized Mesoporous Carbon

**DOI:** 10.3390/s20123365

**Published:** 2020-06-13

**Authors:** Chuhan Lv, Shuangfei Li, Liangxu Liu, Xingyu Zhu, Xuewei Yang

**Affiliations:** 1 Guangdong Technology Research Center for Marine Algal Bioengineering, Guangdong Key Laboratory of Plant Epigenetics, College of Life Sciences and Oceanography, Shenzhen University, Shenzhen 518060, China; LVCHU0510@163.com (C.L.); sfli@szu.edu.cn (S.L.); liangxuliu@icloud.com (L.L.); staruny9562@163.com (X.Z.); 2 Shenzhen Key Laboratory of Marine Biological Resources and Ecology Environment, Shenzhen Key Laboratory of Microbial Genetic Engineering, College of Life Sciences and Oceanography, Shenzhen University, Shenzhen 518055, China; 3 College of Chemistry and Environmental Engineering, Shenzhen University, Shenzhen 518060, China; 4 Longhua Innovation Institute for Biotechnology, Shenzhen University, Shenzhen 518060, China

**Keywords:** mesoporous carbon, carboxylation, glucose oxidase, glucose biosensor

## Abstract

This research revealed the effect of carboxyl-functionalization on the mesoporous carbon (MC)-fixed glucose oxidase (GOx) for promoting the properties of bioelectrodes. It showed that the oxidation time, temperature and concentration, can significantly affect MC carboxylation. The condition of 2 M ammonium persulfate, 50 °C and 24 h was applied in the study for the successful addition of carboxyl groups to MC, analyzed by FTIR. The nitrogen adsorption isotherms, and X-ray diffraction analysis showed that the carboxylation process slightly changed the physical properties of MC and that the specific surface area and pore size were all well-maintained in MC-COOH. Electrochemical characteristics analysis showed that Nafion/GOx/MC-COOH presented better electrocatalytic activity with greater peak current intensity (1.13-fold of oxidation peak current and 4.98-fold of reduction peak current) compared to Nafion/GOx/MC. Anodic charge-transfer coefficients (α) of GOx/MC-COOH increased to 0.77, implying the favored anodic reaction. Furthermore, the GOx immobilization and enzyme activity in MC-COOH increased 140.72% and 252.74%, leading to the enhanced electroactive GOx surface coverage of Nafion/GOx/MC-COOH electrode (22.92% higher, 1.29 × 10^−8^ mol cm^−2^) than the control electrode. Results showed that carboxyl functionalization could increase the amount and activity of immobilized GOx, thereby improving the electrode properties.

## 1. Introduction

Fuel cells, which convert chemical fuels into electricity, have been considered as a renewable power source for electronic equipment. Due to the minimal risk of false-positive responses, enzymes are the most promising bio-receptors used in bio-sensors [1]. Unlike noble metal catalysts which are valuable and rare, enzymes as a biological catalyst possess renewable and abundant sources [2]. Immobilizing redox enzymes (such as glucose oxidase (GOx)) onto electrode material surfaces, has become a keen interest in biofuel cell and sensor development. Anahita Karimi et al. outlined the advantages and limitations of functionalized graphene and graphene-based nanocomposite immobilized enzymes, and proved the possibility of the development of graphene-based enzyme biofuel cells [3]. Michael Holzinger et al. introduced different methods for immobilizing enzymes in carbon nanotubes, summarizing the application and development of carbon nanotubes in enzyme biofuel cells [4]. JingYang et al. Immobilized horseradish peroxidase on a glassy carbon electrode to construct a biocathode [5]. The novel implantable glucose fuel cells (IGFCs) are a potential energy source that can power small electronic devices such as artificial urinary sphincters, cardiac pacemakers, and implanted biosensors [6]. Although biofuel cells (BFCs) work on behalf of a new energy source, it is still tough to apply them commercially. Compared with traditional fuel cells, the application of BFCs suffers from the drawbacks of long-term instability, insufficient power output, and low open-circuit voltage (Ecell) [7]. In particular, enzyme stability is the main reason that restricts the power output of the enzymatic fuel cells [6].

Carbon materials are generally used in electroanalytical investigations due to the low background current, relatively wide potential window, and chemical inertness. Carbon materials are also appropriate for various types of analysis [8] in both analytical and industrial electrochemistry [9]. Mesoporous materials are promising for bio-molecule loading due to the narrow pore size distribution, high specific surface area, and well-ordered pore structures [10]. Large amounts of enzyme were immobilized in nanostructured carbon materials to increase the active surface area of enzymatic bio-electrodes [11]. Nanostructured materials are valid not only for steadying the enzyme activity but also for promoting the capabilities of the nano-biocatalytic systems, such as high enzyme activity, high enzyme loading and improved electron transfer rate. Enzyme immobilization in a mesoporous material could enable further practical applications, as such extending the lifespan of enzyme reactors, increasing the potentiality for recycling the enzymes and improving the power density of biofuel cells [12].

Nano-carbon materials such as graphene [9,13,14] carbon nanotubes (single-wall, multi-wall) [15,16,17], and mesoporous carbon [18,19] are commonly used in immobilizing enzymes and developing enzyme-based electrochemical devices. Mesoporous carbon (MC) obtains properties appropriate for application in biofuel cells and biosensors, including high specific surface areas, high specific pore volume, extremely well-ordered pore structure, tunable pore diameters in the mesopore range and conductivity [20,21]. The most interesting property is that MC can ensure the direct electronic connection of each immobilized matter, enabling electronic penetration [20]. Furthermore, MC obtains additional edge-plane-like deficient sites [22], which are more advantageous for electron transfer to H_2_O_2_ and NADH [23,24]. It makes MC transfer electrons to biomolecules more efficiently, thus contributing to the impressive electrochemical behavior [8]. However, MC also exhibits hydrophobic properties and an unreactive surface on account of the shortage of oxygen-containing functional groups caused by the high carbonization temperature during synthesis [25]. The inferior hydrophobicity and surface reactivity of MC limit the enzyme immobilization and prohibit the access of reactants from the solution to the effective sites of the enzymes on the electrode surface [26].

To further activate the surface of MC and overcome the deficiencies mentioned above, a number of methods that apply strong oxidation agents (nitric acid, ozone, and sulfuric acid) to generate some oxygenated functionalities have been explored. It is known that functional nanomaterials can cause a synergistic effect among biocompatibility, conductivity, and catalytic activity to speed up signal transduction, leading to highly sensitive biosensing [27]. For example, Y. Shin et al. used sulfur-functionalized mesoporous carbon as a heavy-metal sorbent [28]. Guide Yang et al. used nitrogen-functionalized magnetic MC as a sorbent to adsorb Pb (II) and phenol [29]. Ajayan Vinu et al. discussed the effect of different carboxylation conditions on the adsorption and fixation of lysozyme by mesoporous carbon [30]. However, there are few reports on the use of carboxyl-functionalized MC to enhance enzyme activity and electrochemical properties of its related electrodes.

To explore the carboxylic functionalization conditions for MC, oxidation temperature, oxidant concentration, and oxidation time were studied. The functional groups, skeleton structure, pore volume and specific surface area of MC after carboxyl functionalization (MC-COOH) were investigated by Fourier transform infrared (FTIR) spectrometer, wide-angle X-ray diffraction (XRD) diffractometer, and nitrogen adsorption specific surface area analyzer (BET). The influence of-COOH group on the amount and enzyme activity of immobilized GOx was analyzed. Finally, the electrode was prepared using 5% Nafion solution, and the electrochemical properties were analyzed by cyclic voltammetry (CV).

## 2. Materials and Methods

### 2.1. MC Carboxyl Functionalization

MC (pore volume of 0.5 cm^3^ g^−1^, surface area of 150–250 m^2^ g^−1^, 699640, Sigma-Aldrich Corp. St. Louis, MO, USA) was oxidized using an alike process to that reported in the literature [31,32] via oxidation using solutions of ammonium persulfate ((NH_4_)_2_S_2_O_8_, APS) with different concentrations ranging from 0 to 2 M in 2 M sulfuric acid (H_2_SO_4_). In a conventional oxidation experiment, 300 mg of MC was added to 20 mL of the APS solution. To utilize DF-101S heat-collecting magnetic stirrer (Qiuzuo Scientific Instrument Co., Ltd., Shanghai, China), the mixture was stirred at 30 rpm for diverse periods of time up to a maximum of 96 h and at various temperatures ranging from 30 to 80 °C. All the oxidized samples were filtered, washed a few times with distilled water until the washing water was without sulfates, and dried overnight in vacuum drying oven (DZF-6050, Shanghai Yiheng Scientific Instruments Co., Ltd., Shanghai, China).

### 2.2. GOx Immobilization on MC-COOH

The immobilization experiment was conducted by suspending 20 mg of MC-COOH and MC in 1 mL 0.05 M pH 7.0 PBS buffer, which contains 10 mg ml^−1^ GOx (246 U mg^−1^, G8032, Beijing Solarbio Science & Technology Co., Ltd., Beijing, China) at room temperature in a centrifuge tube. The consequent intermixture of GOx solution and the supports was then incubated at 10 °C with agitating at 220 rpm (LYZ-D2403 Superimposed Shaker, Shanghai Longyue Instrument Equipment Co., Ltd., Shanghai, China) for 6 h to attain adsorption equilibrium [30]. After immobilization, the samples were centrifuged (75008800 Medifuge Centrifuge, Thermo Fisher Scientific Co., Ltd., San Jose, CA, USA) at 10,000 rpm for 5 min. The free GOx particles were subsequently separated, and the solid part (GOx/COOH-MC) was washed with PBS buffer 3 times, dried under vacuum using a lyophilizer (triad 2.51, LABCONCO, USA) and stored at −20 °C for further use. In each set of experiments, three biological replicates were performed.

The amount of GOx immobilized on MC and MC-COOH particles was computed by ascertaining the original and terminal concentration of GOx in the liquid solution through ultraviolet spectrophotometry at 450 nm (EPOCH 2 microplate reader, Bio Tek, Winooski, VT, USA). The glucose oxidase activity of GOx/MC-COOH and GOx/MC was analyzed by utilizing a glucose oxidase assay Kit (BC0695, Beijing Solarbio Science & Technology Co., Ltd., Beijing, China). In each set of experiments, three biological replicates were performed.

### 2.3. Fabrication of GOx/MC and GOx/MC-COOH Bioelectrodes

**Figure 1:**
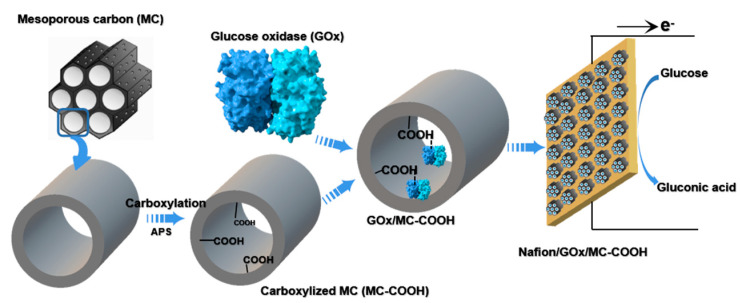
Experimental scheme of mesoporous carbon (MC)-COOH preparation and enzyme immobilization.

The glucose bioelectrode was made through pipetting 20 mg of the dispersions of GOx/MC and GOx/MC-COOH in a pH 7.0 PBS buffer solution with 0.5% Nafion onto carbon cloth as an anode [33]. After drying the Nafion/GOx/MC and Nafion/GOx/MC-COOH-coated carbon cloth electrodes in the air at room temperature, they were rinsed meticulously with pH 7.0 PBS buffer, and stored at 4 °C before use. The experimental scheme was shown as Figure 1.

### 2.4. Characterization of MC-COOH

FTIR spectra of the samples in the 400–4000 cm^−1^ region were acquired on a Nicolet 6700 (USA) spectrometer using the usual KBr pellet technique. XRD analyses were performed on an Empyrean diffractometer (PANalytical B.V., Almelo, The Netherlands) using copper anode radiation over the range of 10°–80°. Nitrogen sorption isotherms were measured with an ASPS 2460 (Micromeritics Instruments Corporation, Norcross, GA, USA) analyzer at 77.4 K. In front of measurement, the powders were degassed at 80 °C under vacuum for more than 6 h.

### 2.5. Electrochemical Measurements of Glucose Bioelectrodes

By using an Autolab PGSTAT 302 N (Switzerland) potentiostat, all electrochemical processes were analyzed. CV was performed in a three-electrode system, which contains a platinum piece counter electrode, an Ag/AgCl electrode, and a working electrode. CV scans were performed at 20–200 mV·s^−1^ scan in the potential range from −1.0 to 1.0 V (v.s. Ag/AgCl) in air-saturated 0.1 M pH 7.0 PBS supplemented with 10 mM glucose at room temperature.

## 3. Results

### 3.1. Effect of Carboxylation Conditions on GOx Adsorption

**Figure 2:**
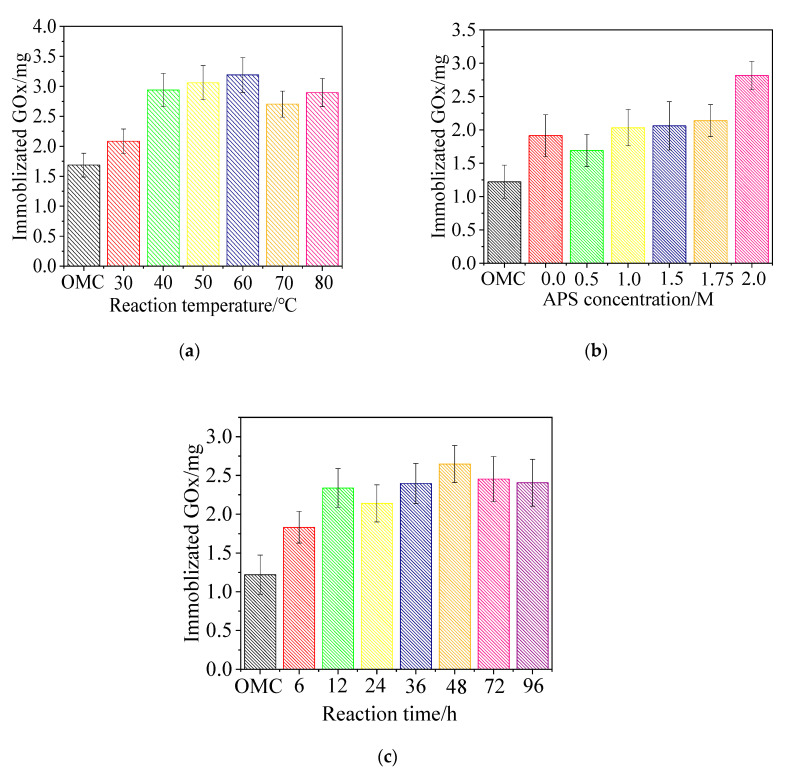
Effects of the carboxylation conditions on the glucose oxidase (GOx) immobilization in carboxylated mesoporous carbon; (**a**) 1.75 M ammonium persulfate (APS), 2 M H_2_SO_4_ at different temperature (30, 40, 50, 60, 70, 80 °C) for 24 h; (**b**) 0, 0.5, 1.0, 1.5, 1.75, 2.0 M APS, 2 M H_2_SO_4_ at 50 °C for 24 h; (**c**) 1.75 M APS and 2 M H_2_SO_4_ at 50 °C for 6, 12, 24, 36, 48, 72, 96 h.

To investigate the influence of carboxylation conditions on GOx immobilization on MC-COOH, various reaction conditions, including different oxidation temperatures, APS concentrations, and reaction times, were investigated, shown as Figure 2. The results showed that the carboxylation condition could significantly influence the GOx immobilization in MC-COOH. Compared with MC, MC-COOH exhibited obvious advantages in enzyme adsorption capacity.

#### 3.1.1. Temperature

As shown in Figure 2a, the carboxylation temperature (30 to 80 °C) could greatly influence the GOx adsorption (increasing from 23.60% to 89.13%) in MC-COOH. As the temperature increased from 30 to 60 °C, the GOx adsorption raised from 2.09 to 3.19 mg GOx/20 mg MC. The GOx absorption of MC-COOH (3.19 mg GOx/20 mg MC) carboxylated at 60 °C increased by 89.13% compared with the unmodified MC (1.69 mg GOx/20 mg MC). However, as the temperature increased from 60 to 80 °C, a decrease in the GOx adsorption (from 3.19 to 2.90 mg GOx/20 mg MC) in MC-COOH was observed. Previous research showed that the oxidation temperature affected the concentration of oxygen-containing groups (acid anhydrides, strongly acidic carboxyl groups, phenols, carbonyls, weakly acidic carboxyl groups, quinones, and lactones) on the surface of the carbon material [34]. It was known that lower temperature profited the alternative formation of acidic MC (containing carboxyl, lactone, lactol, anhydride and phenol) while using APS as the oxidizing agent, whereas higher temperature favored the selective formation of alkalescent MC (containing carbonyl, pyrone, ether and quinone) [35]. Oxidation of mesoporous carbon at lower temperatures will cause oxygen-containing groups to block the pores of the carbon. As the temperature increases, the blocked pores will open, and the specific surface area and total pore volume will be higher than that of carbon materials oxidized at low temperature. Relatively high temperature will cause the total pore volume and specific surface area of the material to be greatly reduced, which is not conducive to adsorption of enzymes [36]. In Figure 2a, it can be seen that the adsorption of mesoporous carbon oxidized at 50 °C and 60 °C were higher. Thus the appropriate temperature was important that it should be high enough to increase the concentration of weakly acidic carboxyl groups, as well as low enough to form the acidic MC [35], which was more favorable to covalent adsorption.

#### 3.1.2. APS Concentration

The amounts of immobilized GOx in MC-COOH with various APS concentrations were presented in Figure 2B. Without adding APS, the GOx adsorption of MC stands at 2.09 mg GOx/20 mg MC. Then, the GOx adsorption rose marginally from 0 M to 1.75 M (2.14 mg GOx/20 mg MC). After that, it rose rapidly, reaching 2.81 mg GOx/20 mg MC at 2 M. Compared with the MC, the sample carboxylated at an APS concentration of 2 M adsorbed 130.47% more GOx. Oxidation with APS was conducive to enhance the concentration of acidic oxygen groups (carboxyl, anhydride and phenol groups). However, oxidation with APS solutions also led to diverse degrees of reduction in pore volume and surface area on the basis of the APS concentration. The porous carbon structure using a higher concentration of APS would be destroyed or collapsed [35], which greatly affected the fixation of GOx. Thus the APS concentration (2 M) was significant to form target chemical groups without excessively destroying the specific surface area and pore size of MC.

#### 3.1.3. Time

Moreover, Figure 2C presented the effects of the MC carboxylation reaction time (6 h to 96 h) on GOx immobilization, which showed the GOx absorption of 48 h (2.65 mg GOx/20 mg MC) and 72 h (2.45 mg GOx/20 mg MC) were largest. In particular, compared to the unmodified MC, the MC samples carboxylated for 48 h and 72 h exhibited 2.17 times and 2.01 times more immobilized enzyme, respectively. The GOx absorption gradually increased from 6 h (1.83 mg GOx/20 mg MC) to 48 h, then declined by 15.88%–3.86% compared to 48 h. During the oxidization process, the concentration of surface lactone groups rose smoothly with time, which was similar to the concentration of carboxyl groups. However, the differences between samples of the concentration of surface lactone groups subjected to different treatment times sharply increased, which meant that the amount grew both by oxidation of the surface phenol groups and by direct formation of lactones. The number of lactone groups changed in correlation with the reaction time, which affected the degree of oxidation of the material [37]. Analyzed by the statistical analysis, MC with 48 h oxidation had significant differences among each group in terms of GOx absorption. Thus the reaction time (48 h) was suitable for the preferable oxidation of MC.

Above all, the results showed that the oxidation temperature, APS concentration, and reaction time could significantly influence MC carboxylation and consequently improve GOx immobilization. From the above experiments, it was found that the adsorption capacity of GOx increased the most (compared with MC, an increase of 130.47%) under 2 M APS, 50 °C and 24 h carboxylation conditions. Thus, the MC oxidized by this condition was selected in the subsequent experiments to prepare electrodes.

### 3.2. Characterization of MC-COOH

#### 3.2.1. Fourier Transform Infrared (FTIR) Spectrum Analysis

**Figure 3:**
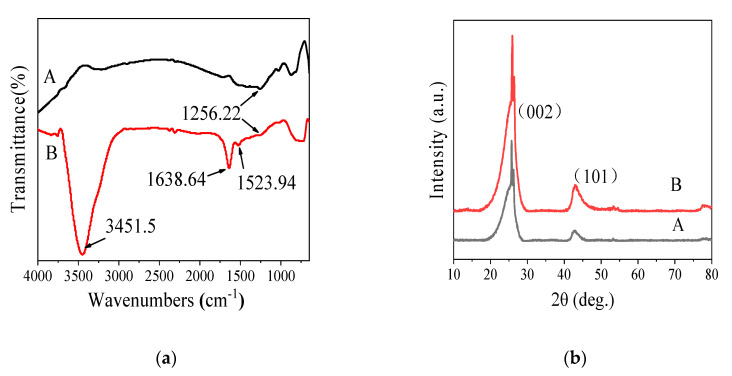
Fourier transform infrared (FT-IR) spectra (**a**) and X-ray diffraction patterns (XRD) (**b**) of MC (A) and MC-COOH (B).

According to the FTIR analysis results, three bands centered at approximately 3451.50 cm^−1^, 1638.64 cm^−1^, and 1523.94 cm^−1^ (Figure 3a, A) were clearly visible in the MC-COOH spectrum, which was not observed in the MC spectrum. The absorption band at 1638.64 cm^−1^ belonged to the stretching frequency of C=O in carboxylic group (O=C−OH) [38,39]. The band at 1523.94 cm^−1^ could be attributed to the symmetric COO- oscillation and/or aromatic ring stretching coupled with greatly conjugated keto groups [31]. The broad peak at 3451.50 cm^−1^ was ascribed to the -OH oscillation [39,40]. The bands at 1256.22 cm^−1^ were observed in both MC-COOH and MC, attributed to C-O-C asymmetric oscillations in ether groups of oxidized mesoporous carbon, which were observed in both MC-COOH and MC [41,42]. These results clearly indicated that -COOH groups were formed on the carbon frameworks by the oxidative treatment.

#### 3.2.2. Wide-Angle X-ray Diffraction (XRD) Analysis

In the wide-angle XRD patterns (Figure 3b), the MC and MC-COOH nanocomposites displayed two extremely sharp peaks centered at approximately 26° and 43°, which corresponded to the (002) and (101) diffractions of graphite [43,44]. The weak diffraction peak around 12.8° (Figure 3b, B) would indicate the presence of graphite oxide [45,46]. It was inferred that the graphitic character of the samples might derive from the highly carbonized MC framework or from the carboxylation reaction [47]. Moreover, the XRD pattern of MC-COOH was similar to that of MC, indicating that there was barely damage to the structure and composition of MC-COOH [32]. Above all, the FTIR and XRD analysis revealed that after functionalization, carboxyl groups were successfully added to MC with a slight change in the skeleton structure.

#### 3.2.3. Nitrogen Adsorption Specific Surface Area (BET) Analysis

**Figure 4:**
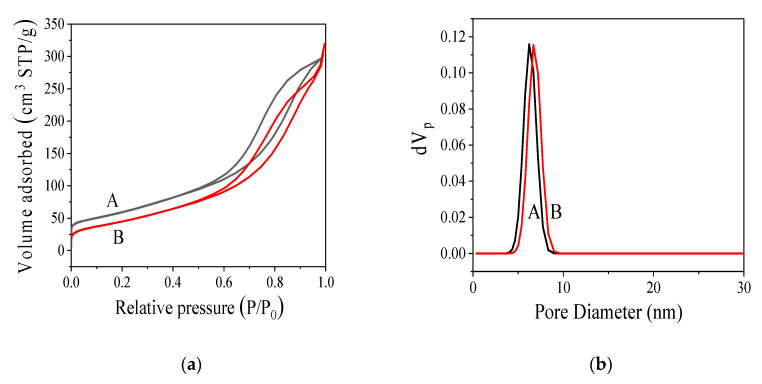
Nitrogen adsorption—desorption isotherms (**a**) and pore size distribution (**b**) of MC (A) and MC-COOH (B).

Nitrogen adsorption/desorption isotherms, and the corresponding pore size distribution curves of the MC and MC-COOH samples were presented in Figure 4. It revealed that the obtained isotherms of two samples (shown as Figure 4a) could rank as type IV isotherms, which exhibited H_4_ hysteresis loops and an obvious capillary condensation step [31,48]. Various isotherms showed distinct hysteresis loops, presenting the characteristics of a porous adsorbent [49]. Type H_4_ hysteresis loops, often did not close before approaching the saturation pressure, were also structured by slit-shaped pores, reflecting the characteristics of activated carbon materials [50]. The hysteresis loop of MC-COOH was situated at P/P_0_ values between 0.6 and 0.8, bespeaking that MC-COOH had an abundant uniform mesoporous structure [40,51].

In addition, the pore size distribution curve based on the adsorption data explicitly identified a limited pore size distribution within 4–12 nm (Figure 4b) and showed that the peak pore sizes of MC (a) and MC-COOH (b) were centered at 6.23 nm, and 6.70 nm, respectively, which allowed them to suitably fix GOx since the threshold pore aperture value to immobilize GOx on nanoporous materials was 3.50 nm [52]. The pore structure parameters of MC and MC-COOH were based on the BET equation to the N_2_ adsorption isotherm at 77 K, as shown in Table 1. Pore size distribution was a significant property with regard to porous adsorbents. The average adsorption pore widths (4 V/SBET) of MC and MC-COOH were acquired to be 7.99 nm and 9.24 nm, separately. Moreover, no significant differences in mesopore volume and total pore volume between MC and MC-COOH were found, indicating that after functionalization, MC-COOH successfully maintained the mesoporous structure. In conclusion, MC-COOH exhibited the appropriate pore size and pore volume for the GOx immobilization.

### 3.3. Direct Electrochemistry of Bioelectrodes Nafion/GOx/MC and Nafion/GOx/MC-COOH

**Figure 5:**
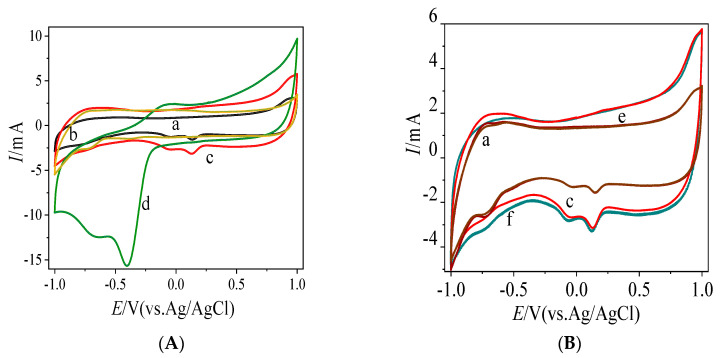

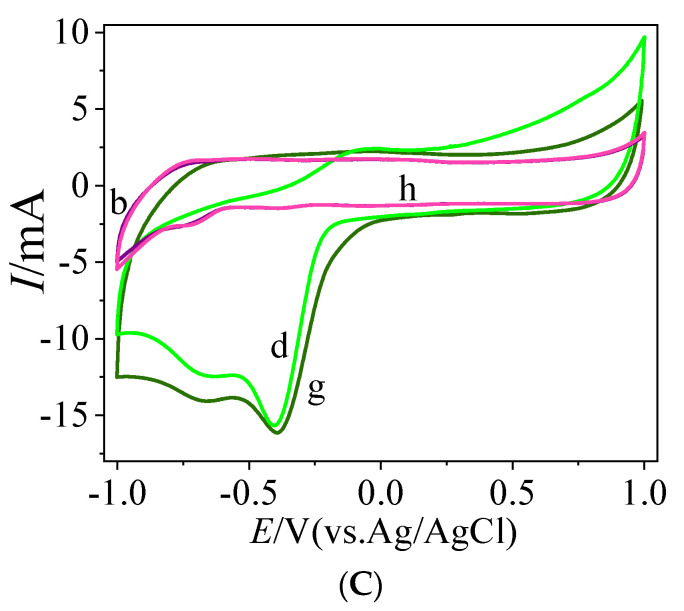
(**A**) Cyclic voltammetry (CV) of MC (a), MC-COOH (b), MC-GOx (c) and COOH-MC-GOx (d) in air-saturated PBS 0.1 M pH 7.0 at scan rate of 50 mV s^−1^ with the addition of 10 mM glucose; (**B**) CV of MC with (a) and without glucose (e), MC-GOx with (c) and without glucose (f) in air-saturated PBS 0.1 M pH 7.0 at scan rate of 50 mV s^−1^; (**C**) CV of COOH-MC with (b) and without glucose (h), COOH-MC-GOx with (d) and without glucose (g) in air-saturated PBS 0.1 M pH 7.0 at scan rate of 50 mV s^−1^.

CV scans of Nafion/MC, Nafion/MC-COOH, Nafion/GOx/MC and Nafion/GOx/MC-COOH were obtained, as shown in Figure 5. In the absence and presence of glucose shown as Figure 5A–C, there is no obvious peak corresponding to the glucose oxidation in the electrode Nafion/MC (a, e) and Nafion/COOH-MC (b, h), indicating that there is no biochemical reaction on the bare MC or COOH-MC to produce electrons [53]. Thus, the currents observed in bioelectrode MC-GOx and COOH-MC-GOx were mainly contributed by the GOx catalysis. As Figure 5A, c, d showed, the oxidation peak current of COOH-MC-GOx was much higher (1.13 times) than MC-GOx. It was known that the direct electron-transfer behavior of the GOx-electrode is as the following [54,55,56]:

GOx (FAD) + Glucose → gluconolactone + GOx (FADH_2_)

As is well known, glucose is the substrate of GOx, whose presence will result in an oxidation reaction and increase the oxidative peak current on electrode surface. Previous studies presented that the redox peak current signal shows the increase due to the electron transfer that occurs between the active sites of glucose oxidase and electrodes [57]. Thus, the GOx content is critical in glucose oxidation. In Table 2, results showed that the amount and enzyme activity of GOx immobilization in the COOH-MC was higher than that of MC-GOx. The total amount of GOx immobilized in COOH-MC was 1.34 times more than that in MC. The increase of the oxidation peak current (280 μA) in COOH-MC is possibly due to the increase of GOx immobilization, thus enhancing the bioactivity of immobilized COOH-MC-GOx.

There were two cathodic peaks observed in the CV curve of the bio-electrode constructed with Nafion/GOx/MC-COOH. In the presence of oxygen, the reduced enzyme is oxidized very quickly at the surface of the electrode [55,56,58]:

GOx-FADH_2_ + O_2_ → GOx-FAD + H_2_O_2_

The catalytic regeneration of the enzyme in its oxidized form causes the loss of reversibility and the increase in size of the reduction peak [58,59,60]. In the air-saturated buffer solution, the shape of CV curve for the direct electron-transfer of GOx showed an increase of reduction peak current, indicating that GOx in the MC and COOH-MC catalyzed the oxygen reduction. Since the main reductive reaction was due to the O_2_ reduction by GOx, the maximum cathodic peak at −0.430 V was speculated to be the peak of reducing oxygen. The similar phenomenon were also observed in other bio-electrodes constructed with GOx as the catalyst. Two peaks appeared in the CV curves of GOD-graphene/PANI/AuNPs modified GCE in an N_2_-saturated PBS [61]. The graphene/polyaniline/Au nanoparticles/glucose oxidase biocomposite modified screen-printed electrode in air-saturated PBS with different glucose concentrations also showed two cathodic peaks [54]. It was reported that the electrode of Nafion-Graphene-GOD/GE exhibited two cathodic peaks in the CV chart under air saturated PBS with different glucose concentrations [56]. Moreover, the electrode constructed by GOx-GMC nanocomposite with 0.13% of GA showed two cathodic peaks in the CV graph scanned under N_2_ saturated PBS [62]. In Figure 5, the CV responses of MPC-CHT-GOx/SPCE and MWCNT-CHT-GOx showed two cathodic peaks in the absence and presence of 3 mM glucose containing O_2_ saturated 0.1 M PBS [63]. However, the reason for the second cathodic peak still stays unclarified. A possible explanation might be that the reduction of the glucose oxidase itself could produce a relatively weak cathodic peak [54,55,56,58,59,64].

**Figure 6:**
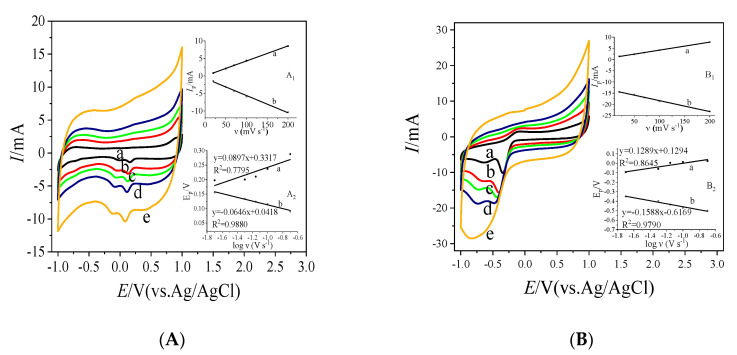
(**A**) CV of MC-GOx at different scan rates (20 mV s^−1^ (a), 50 mV s^−1^ (b), 70 mV s^−1^ (c), 100 mV s^−1^ (d), 200 mV s^−1^ (e)), insets (A_1_ and A_2_) are plots of anodic and cathodic peak currents vs. scan rate and peak potentials vs. log scan rates respectively. (**B**) CV of COOH-MC-GOx at different scan rates (20 mV s^−1^ (a), 50 mV s^−1^ (b), 70 mV s^−1^ (c), 100 mV s^−1^ (d), 200 mV s^−1^ (e)), insets (B_1_ and B_2_) are plots of anodic and cathodic peak currents vs. scan rate and peak potentials vs. log scan rates respectively.

To further study the properties of Nafion/GOx/MC (Figure 6A) and Nafion/GOx/MC-COOH (Figure 6B) in the presence of glucose, CV scanning was performed in air-saturated PBS with 10 mM C_6_H_12_O_6_ with different scan rates. We found that peak potential shifted toward more positive while in reverse toward more negative as the scan rate increased. This phenomenon was typical for irreversible and quasi-reversible systems [65], indicating facile electron transfer kinetics [66]. The good linear relationship between the peak currents versus scan rates [67] (Figure 6, inset A_1_,B_1_) showed that the electron transfer was a surface-controlled electrochemical process [68,69,70] related to the electrode’s electroactive GOx surface coverage (Γ) [71]. It revealed that charge transport was not controlled by diffusion [58], because the dependence of peak currents vs. scan rate barely deviated from linearity [66]. According to [72,73,74], the inter-facial electron transfer on the Nafion/GOx/MC and Nafion/GOx/MC-COOH electrodes were calculated by means of the method of Laviron [75]. Since the ΔE_P_ in the voltammetric curve (Figure 6A) was less than or more than 100 mV (200/*n*, where *n* is the number of electrons transferred), the E_pc_ and E_pa_ linearized in accordance with the logarithm of the scan rates (v), which slopes were −2.3RT/anF and 2.3RT/(1−a)nF, respectively (Figure 6A, inset A_2_, and Figure 6B, inset B_2_). Hence, the anodic charge-transfer coefficients (α) of Nafion/GOx/MC and Nafion/GOx/MC-COOH were calculated to be 0.67 and 0.77. The α increased as the amount of fixed GOx increased, starting with α = 0.67 (GOx concentration was 1.22 mg GOx/20 mg MC) up to approximately α = 0.77 (GOx concentration was 2.81 mg GOx/20 mg MC) [76]. This finding implied that the anodic reaction (glucose oxidation by GOx) was favored.

Additionally, the activity of the GOx immobilized on MC and MC-COOH was investigated (Table 2). The activity of GOx/MC-COOH was 57,216.50 U g^−1^, which was 252.74% higher than the GOx/MC activity. The GOx immobilized on MC-COOH had a unit enzyme activity of 406.60 U mg^−1^, which was 1.65 times greater than the unit enzyme activity of free GOx. The GOx immobilized on MC-COOH exhibited maximal activity. The total charge (Q) of the electrode was calculated from the integration of the CV peaks for estimating the electrical activity of the reductase or oxidase. The surface coverages (Γ) of electroactive GOx in Nafion/GOx/MC and Nafion/GOx/MC-COOH were 1.05 × 10^−8^ mol cm^−2^ and 1.29 × 10^−8^ mol cm^−2^, respectively. The value was calculated according to Γ = Q/nFA, where Q is the charge obtained from the integrals of the anodic peak, *n* is the number of electrons transferred, F is the Faraday constant, and A is the electrode area (2.25 cm^2^) [77]. This value was far more than that of monolayer GOx covering the surface of the bare electrode (2.86 × 10^−12^ mol cm^−2^) [78], which showed that multilayer and three-dimensional GOx coverage were formed on MC and MC-COOH.

## 4. Discussion

### 4.1. Effect of Oxidation Reaction Conditions on the GOx Adsorption

The results showed that the oxidation temperature and time for MC functionalization could significantly influence the amount of enzyme immobilized on MC-COOH. When the oxidative temperature increased from 30 to 60 °C and the oxidative time increased from 6 h to 48 h, the ability of the mesoporous carbon to immobilize GOx increased. This enhanced enzyme adsorption might be due to the remarkable binding affinity of the oxygen-containing groups (mainly COOH groups) on the surface for the functionalized MC [79]. When the oxidation temperature and time further reached a threshold of 60 °C or 48 h, respectively, a huge number of micropores were damaged or blocked, which led to a reduction in GOx immobilization [36]. Interestingly, when the oxidation temperature and time were further raised to 60 °C or 48 h, more GOx immobilization was observed in MC-COOH. A possible explanation was that as the oxidation reaction became harsher, some of the blocked micropores could be opened again and become suitable for enzyme adsorption [36]. Furthermore, for the MC treated with a 2 M APS solution, the amount of GOx adsorbed at 50 °C is 2.81 mg/20 mg MC (Figure 2b), much higher than that of the MC samples treated with other APS concentrations. This result suggested that treatment with a high concentration of APS could introduce more COOH groups at low temperature.

### 4.2. Effect of MC Carboxylation on GOx Immobilization

Compared to those of the enzyme immobilized on MC, the amount and activity of the enzyme immobilized on MC-COOH increased by 130.47% and 252.74%, respectively. The reason may be that the carboxylic groups endow MC-COOH with hydrophilicity and ionic character, thus facilitating the interactions of immobilization. The basal plane itself was electrochemically inert with no such sites ideally contained. GOx was fixed onto the MC-COOH electrode by covalent coupling to construct the enzymatic glucose electrode. Incubation of GOx solution with the MC-COOH electrode led to sufficient contact between the COOH groups of MC and NH_2_ groups on the GOx surface, giving rise to the amide bond (CO-NH) formation [79]. Besides, the apparent reduction in the BET specific surface area and total pore volume of the MC-COOH samples compared to the MC samples was further evidence of the active loading of GOx inside the mesopores because of carboxylation.

### 4.3. Improved Characteristics of the Bioelectrode

The -COOH groups on the carboxylated mesoporous carbon had no effect on the GOx activity or structure, even though these groups could not only physically adsorb GOx but also covalently couple with GOx, thereby increasing the amount of immobilized enzyme and the GOx surface coverage of the enzyme electrode. The results of cyclic voltammetry of Nafion/MC and Nafion/MC-COOH, as shown in Figure 5 (a, b, e, h), show no redox processes occurring with the Nafion/MC and Nafion/MC-COOH electrode in the potential range where the redox peaks of GOx are expected to appear. Shown as the CV curves c and d in Figure 5, the oxidation and reduction peak current increased when the amount of GOx immobilization increased on the modified electrode. The redox peak current signal increased probably due to the electron transfer that occurs between the active sites of glucose oxidase and MC or MC-COOH electrodes [47]. Moreover, the GOx immobilized on MC-COOH presented higher unit enzyme activity of 406.60 U mg^−1^, which was 1.65 times greater than that of GOx immobilized on MC. Thus, the enzyme is not denatured or inactivated, and the glucose oxidation by GOx might be the main reason for producing the anodic peak. The scan rate dependence of the peak currents of the Nafion/GOx/MC and Nafion/GOx/MC-COOH electrodes is plotted in Figure 6 inset A_1_, B_1_. The linear dependence of the peak currents on the scan rate indicates that the redox reaction observed is due to GOx immobilized on the electrode [55]. Thus, it was reasonable to speculate that the enzymatic activity and native conformation had been well retained in the GOx immobilized on MC-COOH [80].

Additionally, the GOx surface coverage of the Nafion/GOx/MC-COOH electrode was calculated to be 22.92%, which was higher than that of the Nafion/GOx/MC electrode. Moreover, this surface coverage was more extensive than that of GOx coated on the surface of a carbon-ceramic electrode (CCE) (1.8 × 10^−9^ mol cm^−2^) [81], MWCNT/PyBA-GOxGA (3.58 × 10^−10^ mol cm^−2^) and MWCNT/PyBA-GOxEDC (1.15 × 10^−9^ mol cm^−2^) [82]. The wide surface area and excellent biocompatibility of MC-COOH and MC increased the absorption of GOx. Therefore, the MC-COOH nanocomposites could provide a beneficial microenvironment for the substantial activity retention and immobilization of GOx. These results demonstrated that the Nafion/GOx/MC-COOH electrode was more advanced for the direct electron transfer of GOx than the Nafion/GOx/MC electrode and some other electrodes [77].

The work of the authors and other references have been compared and shown in the Table 3. Compared with other bioelectrode and biosensors constructed with GOx, our system presented higher anodic peak current and reduction peak current. Moreover, the GOx surface coverage in our system is more than that of other systems. Results indicated that the carboxylized functionalization could enhance the enzyme coverage and activity of the bioelectrode to improve the peak current.

## 5. Conclusions

Electrodes constructed by the immobilization of GOx on MC-COOH exhibited good electrochemical performance. Carboxylated MC was prepared using APS as the oxidant. During this process, oxygen surface groups could be generated, with the pore volume and specific surface area well-maintained. According to single-factor experiments, the conditions of 50 °C, a 2 M APS concentration and 24 h were chosen for the carboxyl addition. Electrochemical characteristics analysis showed that Nafion/GOx/MC-COOH presented better electrocatalytic activity with enhancing peak current intensity (up to 1.13-fold), increasing reduction peak current of O_2_ (up to 4.98 times), rising anodic charge-transfer coefficients (α) (from 0.67 to 0.77), compared to Nafion/GOx/MC. Results showed that carboxylation is a promising strategy for improving the bio-electrode performance through favoring the active catalyst surface and current intensity.

## Figures and Tables

**Table 1:** Pore structure parameters of MC and MC-COOH.

	S_BET_ (m^2^ g^−1^)	V (cm^3^ g^−1^)	W_BJH_ (nm)	W_d_ (nm)	S_BJH_ (m^2^ g^−1^)
MC	226	0.4515	4.42	7.99	202.79
MC-COOH	181	0.4184	4.42	9.24	181.55

S_BET_ represents the BET surface area (S_BET_), the BJH surface area (S_BJH_), single point adsorption total pore volume (V), the BJH volume (V_BJH_), the BJH pore diameter (W_BJH_) and adsorption average pore width (W_d_) (4 V/SBET).

**Table 2:** Enzyme activity of glucose oxidase immobilized in MC and COOH-MC.

Carbon Type	Quality of Immobilized Enzyme (GOx mg/MC g)	Immobilized Enzyme Activity (U/g)	Unit Enzyme Activity U/mg
GOx/MC	61.06	16220.60	265.64
GOx/MC-COOH	140.72	57216.50	406.60

**Table 3:** Comparison between various GOx bioelectrodes.

Electrode	Substrate	Anodic Peak Current	Reduction Peak Current	Quality of Immobilized Enzyme (GOx mg/g)	GOx Surface Coverage	References
Nafion/GOx/MC-COOH	C_6_H_12_O_6_	15.66 mA (50 mv/s)	2.39 mA	140.72	1.29 × 10^−8^ mol cm^−2^	This study
GC/CB/GOx	C_6_H_12_O_6_	0.045 mA	0.036 mA	42		[52]
RGO-AuNPs/PNR/GOx	C_6_H_12_O_6_	0.012 mA	0.006 mA		3.06 × 10^−11^ mol cm^−2^	[72]
GOD/PGR_2_/GCE	C_6_H_12_O_6_	0.009 mA	0.007 mA		2.3 × 10^−10^ mol cm^−2^	[73]
CPE/GOx-SiO_2_/Lig/Fc	C_6_H_12_O_6_	1.10 mA	1.25 mA	25.28		[67]
